# Joint association of chronic pain and sleep patterns with cardiovascular diseases: a prospective study

**DOI:** 10.1186/s12889-025-26145-y

**Published:** 2026-01-16

**Authors:** Lingfang He, Jingfang Yu, Xuerui Wang, Ming Gao, Wei Pan

**Affiliations:** 1 Department of Geriatric Respiratory and Critical Care Medicine, Xiangya Hospital, Central South University, Changsha, China; 2 National Clinical Research Center for Geriatric Disorders, Xiangya Hospital, Central South University, Changsha, 410008 China; 3 Department of Cardiovascular Medicine, Xiangya Hospital, Central South University, 87 Xiangya Road, Changsha, 410008 China; 4 Department of Cardiology, Qilu Hospital of Shandong University, Jinan, Shandong 250012 China

**Keywords:** Chronic pain, Sleep patterns, CVD, Prospective study

## Abstract

**Background:**

Previous studies have analyzed chronic pain and poor sleep behaviors as independent risk factors of cardiovascular diseases (CVD) risk, without mutual adjustment. While chronic pain and poor sleep behaviors frequently co-occurred, the interactive and joint associations of those two factors with CVD risk remains poorly understood.

**Methods:**

A total of 467,689 participants from the UK Biobank were deemed eligible for inclusion in the analysis. The main outcomes were the incidence of CVD and its components including coronary heart disease (CHD) and stroke. Cox proportional hazards model was used to estimate hazard ratios (HRs) and 95% confidence intervals (CIs). First, we assessed the independent associations of chronic pain and sleep patterns with CVD risk, mutually adjusting for these exposures. Then, multiplicative and additive interactions of the different extent of chronic pain and sleep patterns with the CVD risk were examined. Based on the significant multiplicative or additive interaction, the joint association of coexisting the different extent of chronic pain and sleep patterns with the incidence of CVD was further estimated.

**Results:**

During a median follow-up of 16.20 years, 54,743 CVD cases occurred, including 39,478 CHD and 19,696 stroke cases. Chronic pain and sleep patterns were independently associated with increased risk of CVD, CHD and stroke (HR, 1.06–1.83) when they were mutually adjusted. Significant additive interactions existed between chronic localized pain (CLP) and the poor sleep pattern on CVD (relative excess risk due to interaction [RERI], 0.20; 95% CI, 0.02–0.37) and CHD risk (RERI, 0.22; 95% CI, 0.01–0.44). Compared with participants free of chronic pain and having a healthy sleep pattern, those with coexisting chronic widespread pain (CWP) and a poor sleep pattern had a 106% and 105% higher risk of CVD and CHD, respectively.

**Conclusion:**

There were significant additive interactions between CLP and the poor sleep pattern in relation to the risk of CVD and CHD. Among participants experiencing the same extent of chronic pain, there was an increasing trend in the risk of CVD and CHD as sleep patterns deteriorated.

**Supplementary Information:**

The online version contains supplementary material available at 10.1186/s12889-025-26145-y.

## Introduction

Cardiovascular diseases (CVD), including coronary heart disease (CHD) and stroke, are leading causes of death globally [1]. Precisely identifying individuals at high risk for CVD is crucial for its prevention and alleviating the associated disease burden. With population ageing, chronic pain and poor sleep behaviors, two common clinical features of geriatric syndrome, have contributed to an increasing disease burden to both individuals and healthcare systems [2–4]. According to a meta-analysis enrolling 139,933 adult residents in the UK, the prevalence of chronic pain ranged from 35.0% to 51.3% [5]. Data from the UK Biobank showed that 32%, 3%, 33%, 34% and 70% of participants experienced short of long sleep duration, excessive daytime sleepiness, evening chronotype, snoring and insomnia. Rising as two momentous issues in the field of geriatric medicine, chronic pain and poor sleep behaviors have been suggested to be closely related and commonly coexist [6]. According to a systematic review, over 40% of individuals with sleep problems report chronic pain, and more than 50% of individuals with chronic pain report difficulty sleeping [7]. A study enrolling 400 twins showed that the genetic factors influencing sleep quality and chronic pain were highly correlated [8]. Furthermore, previous study basing on genome-wide association study data and using mendelian randomization method demonstrated the bidirectional causal association between chronic pain and sleep disorders [9]. Collectively, the existing evidence from epidemiologic and genetic research suggested that chronic pain and sleep behaviors may interact with each other.

However, previous studies only analyzed chronic pain and poor sleep behaviors as independent risk factors of CVD risk, without mutual adjustment [10–14]. A large prospective study reported that compared with no chronic pain, chronic localized pain (CLP) and chronic widespread pain (CWP) was associated with 14% and 48% increased risk of CVD, respectively [15]. Additionally, Lu qi and his colleagues used five common dimensions of sleep health including chronotype, sleep duration, daytime sleepiness, insomnia and snoring to generate a sleep pattern, and found the risk of CVD in participants with a healthy sleep pattern were significantly lower than those with a poor sleep pattern [16]. There were few studies explored the joint association of chronic pain and sleep behaviors with CVD risk. A comprehensive understanding in the joint associations of coexisting those two common domains may be necessary to identify population at high-risk for CVD and developing primary prevention and treatment strategies in middle-aged and old people.

Thus, to address this problem, we aimed to confirm the independent associations of chronic pain and sleep patterns with CVD risk, with mutual adjustment for these two exposures in multivariable models, systematically assess the additive and multiplicative interactions between chronic pain and sleep patterns on CVD risk, and further estimate the joint association of coexisting chronic pain and sleep patterns with CVD risk.

## Methods

### Study population and design

This research drew upon data from the UK Biobank, which is an extensive, prospective cohort study characterized by its long-term follow-up [17]. Between 2006 and 2010, the UK Biobank recruited approximately 500,000 individuals aged between 40 and 69 from 22 different locations throughout the United Kingdom. After obtaining written informed consent from each participant, the study involved the completion of touchscreen questionnaires and verbal interviews to gather data on demographics, lifestyles, and medical histories. Additionally, participants underwent physical examinations and provided biological samples for further analysis. Information related to participants' health conditions, including mortality, inpatient diagnoses, and surgical procedures was obtained by linking to electronic health records. The UK Biobank has received approval from the West Multi-centre Research Ethics Committee. The present study was conducted under project number 84443.

501,955participants of UK Biobank were available for formal analyses. We excluded 34,266 individuals with CVD at baseline. Ultimately, 467,689 participants free of CVD were included.

### Assessment of chronic pain

In the UK Biobank, information about chronic pain was obtained at baseline using two touchscreen questionnaires (Figure S1). First, participants were posed the question, “In the last month, have you experienced any of the following that interfered with your usual activities?” The response options included pain at seven different body sites (head, face, neck/shoulder pain, back, stomach/abdomen, hip, and knee), “all over the body” or “none of the above”. In this survey, respondents had the option to choose multiple body sites from the seven listed or “all over the body” and those who selected “none of the above” were deemed to be pain-free. Subsequently, individuals who reported experiencing pain were further queried with the question, “Have you had the pain for more than 3 months?” The response options provided were (1) yes or (2) no. Based on the questionnaire findings, we calculated the chronic pain score by summing up the number of sites affected by chronic pain, assigning 1 point for each specific site experiencing chronic pain and 8 points for chronic pain affecting the entire body. Then, participants with the chronic pain score of 0, 1–7, 8 were categorized into three distinct groups: free of chronic pain, CLP and CWP [15].

### Assessment of sleep behaviors and sleep patterns

In this study, we focused on five self-reported of sleep behaviors including chronotype, sleep duration, sleeplessness/insomnia, daytime sleepiness and snoring. The detailed information pertaining to these behaviors was meticulously recorded through touchscreen questionnaires (Table S1).

According to previous studies, early chronotype (definitely a “morning” person or “morning” than “evening” person); sleep 7–8 h/day; free of insomnia (“never/rarely”); no frequent excessive daytime sleepiness (“never/rarely” or “sometimes”) and no snoring were defined as low-risk of sleep behaviors. Those five factors were used to generate a healthy sleep score ranging from 0 to 5 (one point was given for each low-risk sleep behavior). Then, participants were categorized into three groups representing poor, intermediate and healthy sleep patterns corresponding to healthy sleep scores of 0–1, 2–3, 4–5, respectively [18, 19].

## Ascertainment of CVD and death

Within the UK Biobank framework, the electronic inpatient records and death registry data for each participant were obtained through data linkage with several key health information sources including the National Health Service (NHS) Digital for England, Information and Statistics Division for Scotland, and Secure Anonymised Information Linkage for Scotland. The main outcomes of this study were incident CVD and its two principal subtypes—coronary heart disease (CHD) and stroke—over the follow-up period. The criteria for defining CHD and stroke encompassed a range of conditions, including angina pectoris, acute myocardial infarction, subsequent myocardial infarction, specific current complications following acute myocardial infarction, other acute ischemic heart diseases, chronic ischemic heart diseases, subarachnoid hemorrhage, intracerebral hemorrhage, other nontraumatic intracranial hemorrhages, cerebral infarction, stroke (not otherwise specified), occlusion and stenosis of precerebral arteries, occlusion and stenosis of cerebral arteries, other cerebrovascular diseases, cerebrovascular disorders in diseases classified elsewhere, and sequelae of cerebrovascular disease.

### Assessment of covariates

Information of covariates was gathered via touchscreen questionnaires, interview/anthropometric data and diagnosis records. This encompassed demographic details (age, sex, race, Townsend Deprivation Index [TDI]), lifestyle factors (diet, smoking status, alcohol consumption frequency, physical activity) and clinical variables (body mass index [BMI], depression and other psychotic disorders, non-high-density lipoprotein cholesterol [non-HDL], hypertension history and diabetes mellitus history) (Table S2).

### Statistical analyses

Based on a cohort of 467,689 participants free of CVD at baseline, we performed multiple imputation to handle all missing data. We described baseline characteristics of participants according to the extent of chronic pain and sleep patterns. Categorical variables were presented as numbers (percentages, %). Continuous variables were measured as median (interquartile range [IQR]) for abnormal distribution, tested by Quantile–Quantile plot.

In survival analyses, we first used Kaplan–Meier survival curves to compare the survival probability related to chronic pain or sleep patterns. Then, setting follow-up duration as the timescale, Cox proportional hazard models were used to estimate hazard ratios (HRs) and 95% confidence intervals (CIs). Besides covariates including demographic details, lifestyle factors and clinical variables, chronic pain as well as sleep patterns were mutually adjusted to estimate their independent associations with CVD, CHD and stroke risks.

To test multiplicative and additive interactions between chronic pain and sleep patterns in relation to CVD, CHD and stroke, those two exposures were included in Cox proportional hazard models as categorical variables simultaneously, and cross-product terms between the exposures were introduced. Likelihood ratio tests were used to estimate the multiplicative interaction (*P*_interaction_). The additive interaction was tested with 3 indicators including relative excess risk due to interaction (RERI), attributable proportion due to interaction (AP), and synergy index (S) [20]. If any interaction effect was found to be significant, we would further assess the joint association of those two exposures with related outcome events. We categorized the participants into nine mutually exclusive groups based on the combined categories of chronic pain and sleep patterns. Cox proportional hazard models were employed to examine the relationship between these combined groups and the risk of related outcomes, using participants free of chronic pain and having a healthy sleep pattern as the reference group. Schoenfeld residuals method was used to test the proportional hazards assumption, and no evidence suggested deviation from this assumption. The joint effects were examined in three models. In model 1, adjustments were made for age and sex were adjusted. Model 2 additionally included adjustments for race, TDI, diet, smoking status, alcohol consumption, and physical activity. Model 3 further incorporated adjustments for BMI, non-HDL, depression, other psychotic disorders, hypertension and diabetes.

We also performed several sensitivity analyses to test the reliability and robustness of our research. In sensitivity analysis 1, we excluded participants who were diagnosed CVD or died within the first year of follow-up. In sensitivity analysis 2, we further adjusted for analgesic use, with detailed information recorded in the field 20003 and related questionnaires (field 6154). In sensitivity analysis 3, we modified the definition of CVD by excluding only self-reported information.

We used R 4.1.0 and Stata software 16.0 in the study. Two-sided *P*-values < 0.05 indicated a significant threshold.

## Results

The baseline characteristics of participants are presented in Table 1. Among 467,689 individuals included (median age 57.79 years), 206,364 (44.1%) were male, and 442,404 (94.6%) were white. 266,226 (56.9%), 195,546 (41.8%) and 5,917 (1.3%) participants reported free of chronic pain, CLP, and CWP, respectively. Compared with population free of chronic pain, participants with CLP and CWP were more likely to be female and have lower socioeconomic status. They tend to have relatively unhealthy lifestyles, higher BMI, higher level of non-HDL and the history of depression, other psychotic disorders, hypertension and diabetes. They also have poorer sleep patterns. According to sleep patterns, 174,893 (37.4%), 272,160 (58.2%) and 20,636 (4.4%) participants had healthy, intermediate, and poor sleep pattern, respectively. Likewise, individuals with poorer sleep pattern were more likely to be female as well as deprived, and have unhealthy lifestyles, higher BMI, higher non-HDL, and the history of depression, other psychotic disorders, hypertension and diabetes. They also combined by the more severe chronic pain.

**Table 1:** Baseline characteristics

Characteristics	Total	Chronic pain			Sleep patterns		
		Free of chronic pain	CLP	CWP	Healthy	Intermediate	Poor
No. (%) of participants	467689	266226 (56.9)	195546 (41.8)	5917 (1.3)	174893 (37.4)	272160 (58.2)	20636 (4.4)
Age, median (IQR), year	57.79 (50.14, 63.35)	57.65 (49.88, 63.23)	57.95 (50.45, 63.53)	57.74 (51.33, 63.18)	57.41 (49.38, 63.30)	58.06 (50.65, 63.43)	57.13 (50.11, 62.82)
Male, No. (%)	206364 (44.1)	124177 (46.6)	80215 (41.0)	1972 (33.3)	73353 (41.9)	122528 (45.0)	10483 (50.8)
White, No. (%)	442404 (94.6)	252984 (95.0)	184244 (94.2)	5176 (87.5)	166247 (95.1)	257162 (94.5)	18995 (92.0)
TDI, median (IQR)	−2.17 (−3.66, 0.47)	−2.30 (−3.73, 0.16)	−2.02 (−3.57, 0.80)	−0.49 (−2.86, 2.82)	−2.34 (−3.74, 0.11)	−2.10 (−3.63, 0.60)	−1.51 (−3.34, 1.61)
Proper physical activity, No. (%)	359128 (76.8)	208625 (78.4)	146887 (75.1)	3616 (61.1)	140531 (80.4)	204691 (75.2)	13906 (67.4)
Healthy diet, No. (%)	262777 (56.2)	150650 (56.6)	108806 (55.6)	3321 (56.1)	105097 (60.1)	147923 (54.4)	9757 (47.3)
Current smoking, No. (%)	48778 (10.4)	24652 (9.3)	23058 (11.8)	1068 (18.0)	13339 (7.6)	31787 (11.7)	3652 (17.7)
Frequent alcohol consumption, No. (%)	263346 (56.3)	142468 (53.5)	116336 (59.5)	4542 (76.8)	99889 (57.1)	151304 (55.6)	12153 (58.9)
BMI, median (IQR)	26.62 (24.05, 29.75)	26.21 (23.77, 29.11)	27.18 (24.43, 30.56)	28.83 (25.55, 32.86)	25.89 (23.49, 28.75)	26.98 (24.35, 30.16)	28.73 (25.75, 32.44)
Non-HDL, median (IQR)	4.24 (3.55, 4.97)	4.22 (3.54, 4.95)	4.25 (3.56, 5.00)	4.24 (3.52, 5.01)	4.17 (3.51, 4.90)	4.27 (3.58, 5.01)	4.33 (3.59, 5.09)
Depression, No. (%)	73253 (15.7)	32011 (12.0)	38828 (19.9)	2414 (40.8)	21003 (12.0)	46868 (17.2)	5382 (26.1)
Other psychotic disorders, No. (%)	40500 (8.7)	18782 (7.1)	20789 (10.6)	929 (15.7)	11365 (6.5)	26033 (9.6)	3102 (15.0)
Hypertension, No. (%)	113496 (24.3)	58932 (22.1)	52429 (26.8)	2135 (36.1)	35983 (20.6)	70781 (26.0)	6732 (32.6)
Diabetes, No. (%)	20463 (4.4)	10248 (3.8)	9625 (4.9)	590 (10.0)	5949 (3.4)	12874 (4.7)	1640 (7.9)
Chronic pain, No. (%)							
Free of chronic pain	266226 (56.9)	–-	–-	–-	112159 (64.1)	145712 (53.5)	8355 (40.5)
CLP	195546 (41.8)	–-	–-	–-	61630 (35.2)	122455 (45.0)	11461 (55.5)
CWP	5917 (1.3)	–-	–-	–-	1104 (0.6)	3993 (1.5)	820 (4.0)
Sleep patterns, No. (%)							
Healthy	174893 (37.4)	112159 (42.1)	61630 (31.5)	1104 (18.7)	–-	–-	–-
Intermediate	272,160 (58.2)	145712 (54.7)	122455 (62.6)	3993 (67.5)	–-	–-	–-
Poor	20636 (4.4)	8355 (3.1)	11461 (5.9)	820 (13.9)	–-	–-	–-

*CLP* Chronic localized pain, *CWP* Chronic widespread pain, *IQR* Interquartile range, *TDI* Townsend deprivation index, *BMI* Body mass index

During a median follow-up of 16.2 years, 54,743 CVD cases occurred, including 39,478 CHD and 19,696 stroke cases. Kaplan–Meier curves stratified by the extent of chronic pain and sleep patterns are presented in Figure S2. These curves demonstrate that participants with CWP or the poor sleep pattern exhibited higher risks of CVD, CHD and stroke. Table 2 shows the independent and mutually adjusted associations of chronic pain and sleep patterns with incident CVD, CHD and stroke. Compared with participants free of chronic pain, those with CLP and CWP had a 23% (95% CI: 1.21–1.25) and 76% (95% CI: 1.66—1.86) higher risk of CVD. Compared with the healthy sleep pattern, intermediate and poor sleep pattern-related HRs for CVD were 1.11 (95% CI: 1.09–1.13) and 1.24 (95% CI: 1.20–1.29). Likewise, we also observed significant relationships between chronic pain as well as sleep patterns and CHD as well as stroke risks.

**Table 2:** HRs* for CVD by chronic pain and sleep patterns with adjustment for each other

Exposures		CVD		CHD		Stroke	
		HR (95% CI)	*P*	HR (95% CI)	*P*	HR (95% CI)	*P*
Chronic pain	Free of chronic pain	Reference		Reference		Reference	
	CLP	1.23 (1.21, 1.25)	< 0.001	1.26 (1.24, 1.29)	< 0.001	1.17 (1.14, 1.21)	< 0.001
	CWP	1.76 (1.66, 1.86)	< 0.001	1.83 (1.72, 1.96)	< 0.001	1.58 (1.43, 1.74)	< 0.001
Sleep patterns	Healthy	Reference		Reference		Reference	
	Intermediate	1.11 (1.09, 1.13)	< 0.001	1.13 (1.11, 1.16)	< 0.001	1.06 (1.03, 1.10)	< 0.001
	Poor	1.24 (1.20, 1.29)	< 0.001	1.25 (1.20, 1.31)	< 0.001	1.21 (1.14, 1.30)	< 0.001

*CVD* Cardiovascular diseases, *CHD* Coronary heart disease, *CLP* Chronic localized pain, *CWP* Chronic widespread pain, *HR* Hazard ratio, *CI* Confidence interval

^*^Adjusted for age, sex, race, Townsend deprivation index, smoking status, alcohol consumption, diet, physical activity, BMI, non-HDL, depression, other psychotic disorders, hypertension, diabetes mellitus history and sleep patterns/chronic pain

**Fig. 1:**
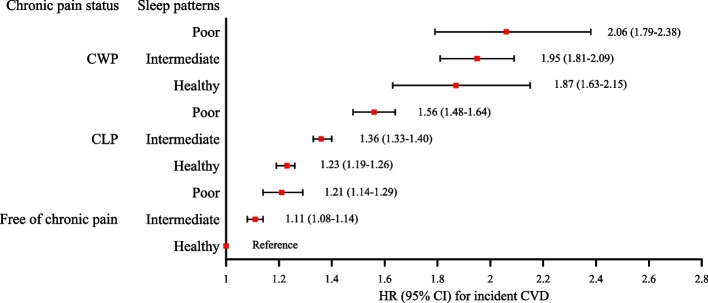
The joint association of chronic pain and sleep patterns with CVD risk

**Fig. 2:**
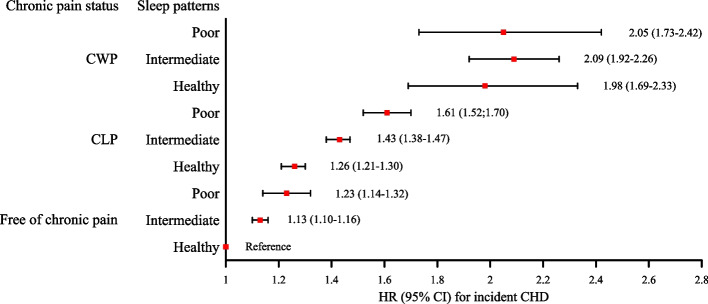
The joint association of chronic pain and sleep patterns with CHD risk

Later, we investigated interactions and joint associations of chronic pain and sleep patterns with CVD outcomes. Significant full-adjusted additive interactions were observed for CLP and the poor sleep pattern in relation to CVD and CHD risks (RERI 0.20, 95% CI: 0.02–0.37; RERI 0.22, 95% CI: 0.01–0.44) (Table 3). In comparison with individuals without chronic pain and having a healthy sleep pattern, those with coexisting CWP and a poor sleep pattern had the highest risks for CVD and CHD, the HRs were 2.06 (95% CI: 1.79–2.38) and 2.05 (95% CI: 1.73–2.42), respectively, in model3 (Figs. 1 and 2, Table S3). The results of 3 sensitivity analyses were consistent with main analyses (Table S4 and S5).

**Table 3:** Additive and multiplicative interactions between chronic pain as well as sleep patterns and CVD outcomes

		Additive interaction (95% CI)			Multiplicative interaction
Outcomes	Exposures	RERI (95% CI)	AP (95% CI)	S (95% CI)	*P interaction*
CVD	CLP and intermediate sleep pattern	0.03 (−0.01, 0.07)	0.02 (−0.01, 0.05)	1.09 (0.95, 1.23)	0.566
	CLP and poor sleep pattern	**0.20 (0.02, 0.37)**	**0.09 (0.02, 0.16)**	**1.20 (1.04, 1.39)**	
	CWP and intermediate sleep pattern	−0.05 (−0.26, 0.17)	−0.03 (−0.19, 0.13)	0.90 (0.52, 1.55)	
	CWP and poor sleep pattern	−0.02 (−0.41, 0.38)	0.01 (−0.20, 0.18)	0.98 (0.68, 1.42)	
CHD	CLP and intermediate sleep pattern	0.04 (−0.01, 0.09)	0.03 (−0.01, 0.07)	1.11 (0.96, 1.27)	0.437
	CLP and poor sleep pattern	**0.22 (0.01, 0.44)**	**0.10 (0.01, 0.18)**	**1.20 (1.02, 1.41)**	
	CWP and intermediate sleep pattern	−0.05 (−0.30, 0.21)	−0.03 (−0.22, 0.15)	0.90 (0.50, 1.62)	
	CWP and poor sleep pattern	−0.16 (−0.63, 0.30)	−0.08 (−0.32, 0.16)	0.86 (0.57, 1.31)	
Stroke	CLP and intermediate sleep pattern	0.03 (−0.04, 0.10)	0.02 (−0.03, 0.08)	1.13 (0.82, 1.56)	0.839
	CLP and poor sleep pattern	0.12 (−0.13, 0.36)	0.06 (−0.06, 0.19)	1.16 (0.87, 1.56)	
	CWP and intermediate sleep pattern	−0.07 (−0.40, 0.26)	−0.06 (−0.33, 0.22)	0.79 (0.23, 2.72)	
	CWP and poor sleep pattern	0.23 (−0.40, 0.86)	0.11 (−0.17, 0.39)	1.28 (0.66, 2.48)	

Referent sleep group: the healthy sleep pattern; Referent pain group: free of chronic pain; *RERI* relative excess risk due to interaction, *AP* proportion of disease attributable to interaction, *S* synergy index, *CI* confidence interval, *CVD* cardiovascular disease, *CHD* coronary heart disease, *CLP* chronic localized pain, *CWP* chronic widespread pain

## Discussion

In this study, participants with a poor sleep pattern had higher proportions of coexisting CLP and CWP, compared to those with the healthy sleep pattern. Both chronic pain and sleep patterns were independently associated with higher risks of CVD, CHD and stroke. Significant additive interactions were observed between CLP and a poor sleep pattern in relation to CVD and CHD. The coexistence of CLP and a poor sleep pattern was related to a 1.20-fold increased risk of CVD and CHD, with 9% and 10% of excess risk attributable to the additive interaction, respectively. Compared to participants with no chronic pain and a healthy sleep pattern, those with CWP combined with a poor sleep pattern had a 1.06- and 1.05-fold increased risks incident CVD and CHD, respectively.

Based on baseline data, we found that participants with chronic pain were more likely to report the poor sleep pattern, consistent with findings from previous studies. According to a meta-analysis, the prevalence of overall sleep disorders among people with chronic pain was 44%. And individuals with chronic pain were almost 13 times more likely to be diagnosed with insomnia [21]. Considering the multi-dimension of sleep, we used the definition of sleep pattern to describe the whole sleep condition and found that 9.5% participants with CWP had a poor sleep pattern, far beyond the free of chronic pain prevalence in individuals with a poor sleep pattern (2.5%). These findings indicated a close relationship between chronic pain and poor sleep characteristics.

Upon reviewing the existing literature, we observed that majority of prior studies investigating the association between chronic pain, sleep characteristics and CVD were conducted without mutually adjusting for the two factors [16, 22]. Only a few studies exploring the association between chronic pain and CVD or other health outcomes have adjusted sleep characteristics as confounding factors. A previous observational study involving 1,609 participants found that CWP was associated with a higher risk of all-cause mortality (HR: 1.95, 95% CI: 1.26–3.03) [23]. However, after adjusting for confounding factors including poor sleep characteristics and smoking, the association became statistically non-significant. Even though this change was not solely due to the adjustment for sleep characteristics, it suggests that sleep factors may act as confounders or mediators in the association between chronic pain and the risk of all-cause mortality. In contrast to this finding, another study involving 1,091 elderly community residents found that higher pain frequency (≥ 2 times/week), severe pain intensity, poor pain experience, and higher pain indices (calculated based on pain frequency, location, and intensity) were associated with an increased risk of CVD incidence compared to participants without a history of pain. These associations remained significant even after adjusting for factors such as sleep duration and sleep quality [24]. Similar to the results of this study, in our study, after mutually adjusting for each other, chronic pain and sleep patterns were independently associated with a higher risk of CVD and CHD incidence, respectively, suggesting that chronic pain and the poor sleep pattern are independent risk factors for CVD and CHD.

Although existing studies indicated that poor sleep characteristics and chronic pain often coexist and interact, with overlapping genetic variations, few studies have explored their interactive effects on health outcomes. In light of their close associations, we explored the multiplicative and additive interactions of chronic pain and sleep patterns on CVD. Our results suggested that there was a synergistic effect of CLP and the poor sleep pattern on the risk of CVD and CHD, and additive interaction accounted for 36% (0.20/0.56) and 36% (0.22/0.61) of CVD and CHD events, respectively. This means that by controlling for either poor sleep patterns or CLP, nearly 36% of CVD and CHD cases might be preventable. These findings indicated that the excessive risk of CVD and CHD depend partly on the coexistence of chronic pain and poor sleep pattern, suggesting the importance of the healthy sleep pattern in populations with chronic pain to reduce the burden of CVD, and conversely, emphasizing the importance of preventing chronic pain conditions in populations with the poor sleep pattern. Against the backdrop of an aging population and the high prevalence of chronic pain and poor sleep health, our findings may offer a novel perspective for the prevention of CVD. Considering that chronic pain is closely related to lower physical activity and higher risk of depression, previous studies reporting the synergistic effects of poor sleep characteristics, lower physical activity, and depression on CVD or mortality also supported our findings in some extent [8, 25, 26]. Our team used the UK Biobank to assess the joint effects of sleep duration and depression on the risk of CMDs and mortality, finding that short sleep duration combined with depression had a significant synergistic effect on the risk of CHD and all-cause mortality, with 56% and 49% all-cause death and CHD cases attributable to the coexistence of short sleep duration and depression [27]. In another study also based on the UK Biobank, Bo-Huei and colleagues explored the joint effects of sleep patterns and physical activity on the risk of mortality, finding that lower physical activity may amplify the negative effects of poor sleep patterns on the risk of mortality [28]. Similarly, in this study, we observed an increasing trend in the risk of CVD and CHD with worsening sleep patterns among participants with the same chronic pain status. These findings may offer robust evidence to support the enhancement of comprehensive assessments aimed at identifying populations with both chronic pain and poor sleep behaviors, thereby facilitating primary prevention strategies for CVD.

This study has several strengths. First, leveraging the UK Biobank's large-scale, longitudinal cohort design, we can implement a “3 × 3” classification framework to stratify chronic pain status and sleep patterns with high statistical power, enabling nuanced subgroup analyses. Second, this study comprehensively assessed the joint association of chronic pain and sleep patterns with CVD for the first time. The results may supply key evidence to guide the identification of high-risk groups for CVD, enabling targeted prevention strategies. There are still limitations in this study. First, as an observational study, our results may be inevitably affected by residual confounding factors and reverse causation. Although, to mitigate this potential bias, we excluded participants diagnosed with CVD within one year of follow-up in the sensitivity analysis, and the results remained largely unchanged. Secondly, over 90% of the participants in this cohort were of White ethnicity, and previous studies have documented a “healthy volunteer bias” that may limit the generalizability of our findings [29]. Thus, caution is warranted when extrapolating our findings to populations from other countries or racial/ethnic groups. Third, although sleep patterns, chronic pain status, and covariates were assessed at baseline, participant behaviors may have changed during the extended follow-up period, potentially introducing time-varying confounding. Finally, self-reported measures were employed for variable assessment, which may introduce measurement bias due to recall inaccuracies, social desirability effects, or misinterpretation of questionnaire items.

## Conclusions

In conclusion, our results showed that significant additive interaction existed between CLP as well as the poor sleep pattern in relation to the risk of CVD and CHD. Among participants experiencing the same extent of chronic pain, there was an increasing trend in the risk of CVD and CHD as sleep patterns deteriorated. Our findings provided epidemiological evidence that underscores the importance of adopting a holistic approach to manage both chronic pain and sleep patterns as a primary preventive measure against among middle-aged and elderly people.

## Supplementary Information

Supplementary Material 1.

## Data Availability

The UK Biobank resource is available to researchers who wish to access the data by completing the registration form in the UK Biobank Access Management System ([https://ams.ukbiobank.ac.uk/ams/](https:/ams.ukbiobank.ac.uk/ams)).

## Abbreviations

CVD: Cardiovascular diseases
CHD: Coronary heart disease
CLP: Chronic localized pain
CWP: Chronic widespread pain
TDI: Townsend Deprivation Index
IQR: Interquartile range
RERI: Relative excess risk due to interaction
AP: Attributable proportion
S: Synergy index
