# Supplementary material for: Joint association of chronic pain and sleep patterns with cardiovascular diseases: a prospective study
Source: BMC Public Health. 2026 Jan 16;26:565. doi: 10.1186/s12889-025-26145-y (PMC12896144; doi:10.1186/s12889-025-26145-y)
Supplement: Supplementary file 1 — Supplementary Material 1. [file 12889_2025_26145_MOESM1_ESM.pdf]

Table S1: Five self-reported dimensions of sleep.

Five dimensions of sleep	Field ID	Question phrasing	Answer options
Sleep duration	1160	About how many hours' sleep do you get in every 24 hours? (please include naps)	Ordinal (1 to 23 hours, in 1-hour increments)
Chronotype	1180	Do you consider yourself to be: definitely a morning person, more a morning than evening person, more an evening than a morning person, or definitely an evening person?	Ordinal (1 = Definitely a morning person; 2 = More a morning than evening person; 3 = More an evening than a morning person; 4 = Definitely an evening person)
Sleeplessness/insomnia	1200	Do you have trouble falling asleep at night or do you wake up in the middle of the night?	Ordinal (0 = Never/rarely; 1 = Sometimes; 2 = Usually)
Snoring	1210	Does your partner or a close relative or friend complain about your snoring?	Ordinal (0 = No; 1 = Yes)
Excessive Daytime dozing/sleeping	1220	How likely are you to doze off or fall asleep during the daytime when you don't mean to? (e.g. when working, reading or driving)	Ordinal (0 = Never/rarely; 1 = Sometimes; 2 = Often; 3 = All of the time)

Table S2. Definitions of confounding variables

Variables	Definition
Age	Continuous variable
Sex	Male and female
Race	White and non-white
Townsend deprivation index	This indicator reflected the integrated condition of housing, employment, and social class individually
Physical activity	Proper physical activity was defined as engagement in at least 150 minutes of walking, moderate activity per week, or 75 minutes of vigorous activity. Data-Field: 22036
Smoking	Current smoking and non-current smoking. Data-field: 20116
Alcohol consumption	Excessive drinking was defined ≥3 times per week. Data-field: 1558
Diet	Total fruit and vegetable intake: >4.5 pieces or servings a week. Total fish intake: >2 per week. Processed and red meat intake: 2 or fewer times intake of processed meat per week and 5 or fewer times intake of red meat per week. Healthy diet defined as ≥2 of the healthy food items. Data-field: 1289, 1299, 1309, 1319, 1329, 1339, 1349, 1369, 1379, 1389
Body mass index	Weight in kilograms (kg) divided by the square of the height in meters (m^2). Data-field: 21001
Non-high-density lipoprotein cholesterol	Continuous variable. The absolute difference of blood cholesterol and high-density lipoprotein cholesterol. Data-field: 30690, 30760
Depression and other psychotic disorders	Including depression and other psychotic disorders. Data-field: 2050, 2060, 2090, 2100, 20126, 4598, 4609, 4620, 4631, 4642, 4653, 5375, 5386, 5663, 5674, 6156, 20002 (1286, 1289, 1291), 20003, 41270 (F32, F20-F25, F28-F31, F34, F38, F39)

Diabetes mellitus	Including insulin-dependent, non-insulin-dependent, malnutrition-related, other specific, and unspecified diabetes mellitus. Data-field: 130707, 130709, 130711, 130713, 130715
Hypertension	Including essential hypertension, hypertensive heart disease, hypertensive renal disease, hypertensive heart and renal disease, and second hypertension. Data-field: 131287, 131289, 131291, 131293, 131295

Data-field: A data-field is the fundamental block of data held within the UK Biobank repository and identifies the results of a single question, measurement or result (or part thereof). For example, Data-field 22036 means the information "At or above moderate/vigorous/walking recommendation" can be accessed from https://biobank.ndph.ox.ac.uk/showcase/field.cgi?id=22036.

Table S3. Joint associations of chronic pain and sleep patterns with CVD and CHD in two models, respectively.

Outcomes	Chronic pain	Sleep patterns	Model 1		Model 2	
			HR (95% CI)	*P*	HR (95% CI)	*P*
CVD	Free of chronic pain	Healthy	Reference		Reference	
		Intermediate	1.19 (1.16, 1.22)	< 0.001	1.16 (1.13, 1.19)	< 0.001
		Poor	1.45 (1.36, 1.54)	< 0.001	1.36 (1.28, 1.45)	< 0.001
	CLP	Healthy	1.32 (1.28, 1.36)	< 0.001	1.28 (1.24, 1.32)	< 0.001
		Intermediate	1.61 (1.57, 1.65)	< 0.001	1.51 (1.48, 1.55)	< 0.001
		Poor	2.11 (2.02, 2.22)	< 0.001	1.89 (1.81, 2.43)	< 0.001
	CWP	Healthy	2.40 (2.09, 2.76)	< 0.001	2.12 (1.84, 2.43)	< 0.001
		Intermediate	2.81 (2.62, 3.02)	< 0.001	2.37 (2.21, 2.54)	< 0.001
		Poor	3.43 (2.97, 3.96)	< 0.001	2.76 (2.39, 3.18)	< 0.001
CHD	Free of chronic pain	Healthy	Reference		Reference	
		Intermediate	1.22 (1.18, 1.26)	< 0.001	1.19 (1.16, 1.23)	< 0.001
		Poor	1.51 (1.40, 1.62)	< 0.001	1.42 (1.32, 1.53)	< 0.001
	CLP	Healthy	1.36 (1.31, 1.41)	< 0.001	1.33 (1.28, 1.37)	< 0.001
		Intermediate	1.71 (1.66, 1.76)	< 0.001	1.62 (1.57, 1.66)	< 0.001
		Poor	2.26 (2.14, 2.38)	< 0.001	2.02 (1.91, 2.14)	< 0.001
	CWP	Healthy	2.61 (2.22, 3.06)	< 0.001	2.28 (1.95, 2.86)	< 0.001
		Intermediate	3.13 (2.89, 3.39)	< 0.001	2.63 (2.42, 2.85)	< 0.001
		Poor	3.60 (3.05, 4.25)	< 0.001	2.87 (2.43, 3.39)	< 0.001

Model 1: Adjusting for age and sex;

Model 2: Further adjusting for race, Townsend deprivation index, smoking status, alcohol consumption, diet and physical activity.

CVD: cardiovascular disease; CHD: coronary heart disease; CLP: chronic localized pain; CWP: chronic widespread pain; HR: Hazard ratio; CI: Confidence interval.

Table S4. Sensitivity analyses of additive and multiplicative interactions between chronic pain as well as sleep patterns and CVD

		Additive interaction (95% CI)			Multiplicative interaction
Sensitivity analyses	Exposures	RERI (95% CI)	AP (95% CI)	S (95% CI)	*P interaction*
Sensitivity analyses 1	CLP and intermediate sleep pattern	0.02 (-0.02, 0.07)	0.02 (-0.02, 0.05)	1.07 (0.94, 1.22)	0.592
	CLP and poor sleep pattern	0.19 (0.01, 0.37)	0.09 (0.02, 0.16)	1.20 (1.03, 1.39)	
	CWP and intermediate sleep pattern	-0.04 (-0.26, 0.18)	-0.03 (-0.19, 0.14)	0.91 (0.52, 1.59)	
	CWP and poor sleep pattern	-0.02 (-0.42, 0.39)	-0.01 (-0.21, 0.19)	0.98 (0.67, 1.44)	
Sensitivity analyses 2	CLP and intermediate sleep pattern	0.03 (-0.01, 0.07)	0.02 (-0.01, 0.06)	1.12 (0.95, 1.31)	0.342
	CLP and poor sleep pattern	0.19 (0.03, 0.36)	0.09 (0.02, 0.16)	1.22 (1.05, 1.41)	
	CWP and intermediate sleep pattern	-0.12 (-0.31, 0.07)	-0.10 (-0.27, 0.07)	0.67 (0.31, 1.44)	
	CWP and poor sleep pattern	-0.01 (-0.38, 0.37)	-0.01 (-0.19, 0.19)	1.00 (0.69, 1.45)	
Sensitivity	CLP and	0.03 (-0.01, 0.07)	0.02 (-0.01, 0.05)	1.09 (0.95, 1.24)	0.569

analyses 3	intermediate sleep pattern			
	CLP and poor sleep pattern	0.19 (0.01, 0.36)	0.09 (0.01, 0.16)	1.19 (1.03, 1.38)
	CWP and intermediate sleep pattern	-0.05 (-0.26, 0.17)	-0.03 (-0.19, 0.13)	0.90 (0.52, 1.54)
	CWP and poor sleep pattern	-0.05 (-0.44, 0.35)	-0.02 (-0.22, 0.17)	0.96 (0.66, 1.39)

Sensitivity analysis 1: Excluding participants who were diagnosed CVD or died within the first year of follow-up;

Sensitivity analysis 2: Further adjusting for analgesics;

Sensitivity analysis 3, Modifying the definition of CVD by excluding only self-reported information.

Referent sleep group: the healthy sleep pattern; Referent pain group: free of chronic pain; RERI: relative excess risk due to interaction; AP: proportion of disease attributable to interaction; S: synergy index; CI: confidence interval; CVD: cardiovascular disease; CHD: coronary heart disease; CLP: chronic localized pain; CWP: chronic widespread pain.

Table S5. Sensitivity analyses of joint association of chronic pain and sleep patterns with CVD

Sensitivity analyses	Chronic pain	Sleep patterns	HR (95% CI)	*P*
Sensitivity analyses 1	Free of chronic pain	Healthy	Reference	
		Intermediate	1.11 (1.08, 1.14)	< 0.001
		Poor	1.21 (1.13, 1.29)	< 0.001
	CLP	Healthy	1.23 (1.19, 1.27)	< 0.001
		Intermediate	1.36 (1.32, 1.39)	< 0.001
		Poor	1.55 (1.48, 1.63)	< 0.001
	CWP	Healthy	1.86 (1.61, 2.14)	< 0.001
		Intermediate	1.94 (1.81, 2.09)	< 0.001
		Poor	2.05 (1.77, 2.37)	< 0.001
Sensitivity analyses 2	Free of chronic pain	Healthy	Reference	
		Intermediate	1.09 (1.06, 1.12)	< 0.001
		Poor	1.21 (1.13, 1.29)	< 0.001
	CLP	Healthy	1.17 (1.13, 1.20)	< 0.001
		Intermediate	1.29 (1.25, 1.32)	< 0.001
		Poor	1.48 (1.41, 1.56)	< 0.001
	CWP	Healthy	1.79 (1.55, 2.06)	< 0.001
		Intermediate	1.76 (1.63, 1.90)	< 0.001
		Poor	1.93 (1.66, 2.25)	< 0.001
Sensitivity analyses 3	Free of chronic pain	Healthy	Reference	
		Intermediate	1.11 (1.08, 1.14)	< 0.001
		Poor	1.22 (1.14, 1.30)	< 0.001
	CLP	Healthy	1.23 (1.19, 1.27)	< 0.001
		Intermediate	1.37 (1.33, 1.40)	< 0.001
		Poor	1.56 (1.49, 1.64)	< 0.001
	CWP	Healthy	1.89 (1.64, 2.17)	< 0.001
		Intermediate	1.96 (1.82, 2.10)	< 0.001
		Poor	2.06 (1.78, 2.38)	< 0.001

Sensitivity analysis 1: Excluding participants who were diagnosed CVD or died within the first year of follow-up;

Sensitivity analysis 2: Further adjusting for analgesics;

Sensitivity analysis 3, Modifying the definition of CVD by excluding only self-reported information.

CVD: cardiovascular disease; CHD: coronary heart disease; CLP: chronic localized pain; CWP: chronic widespread pain; HR: Hazard ratio; CI: Confidence interval.

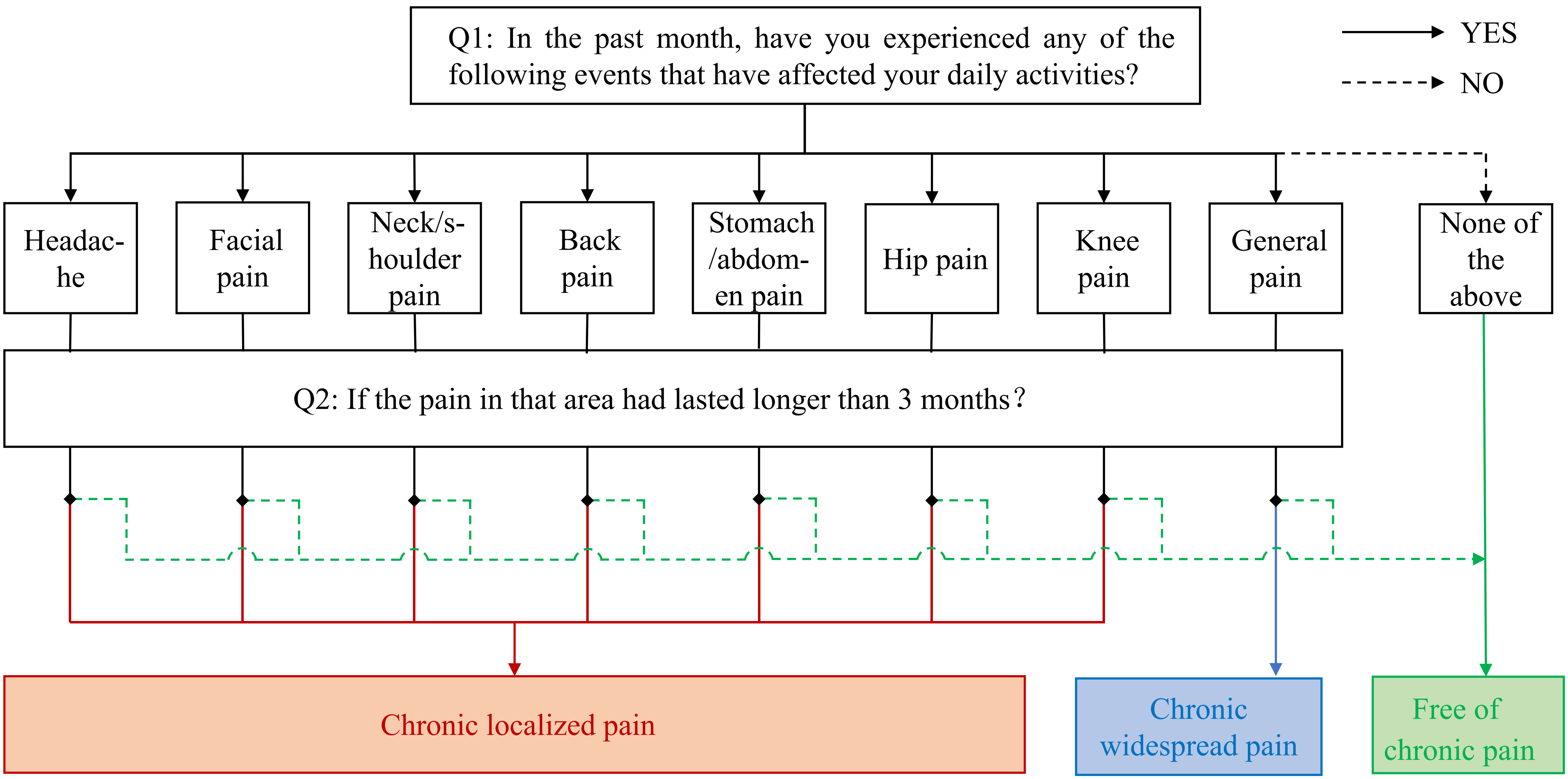

Figure S2. Definition of chronic pain

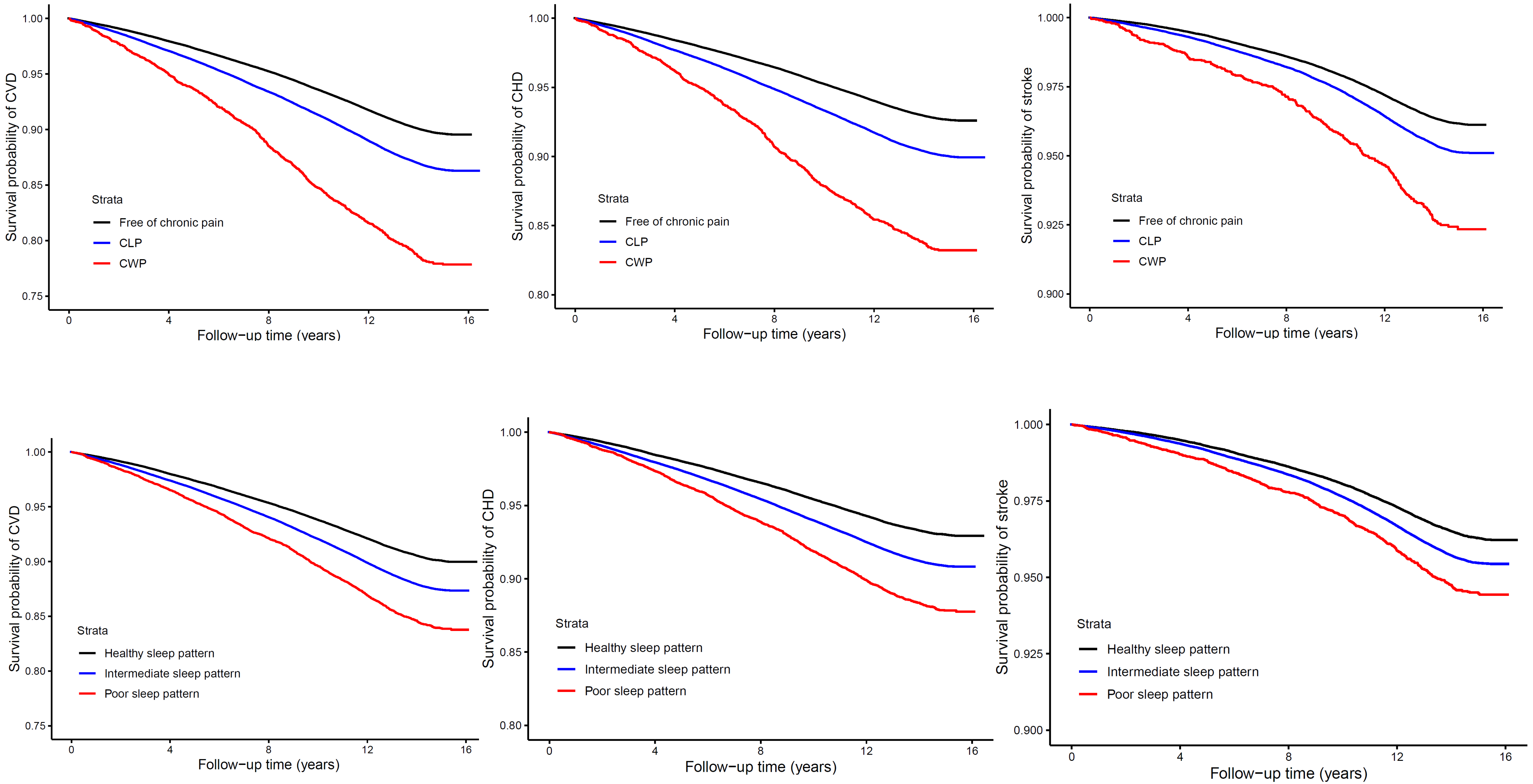

Figure S3. Kaplan-Meier curves
